# Moving, fast and slow: Behavioural insights into bradykinesia in Parkinson’s disease

**DOI:** 10.1093/brain/awad069

**Published:** 2023-03-02

**Authors:** Damian M. Herz, Peter Brown

**Affiliations:** 1 MRC Brain Network Dynamics Unit at the University of Oxford, Nuffield Department of Clinical Neurosciences, University of Oxford, OX1 3TH, Oxford, United Kingdom; 2 Movement Disorders and Neurostimulation, Department of Neurology, Focus Program Translational Neuroscience (FTN), University Medical Center of the Johannes Gutenberg-University Mainz, 55131 Mainz, Germany

**Keywords:** vigour, optimal motor control, utility theory, dopamine

## Abstract

The debilitating symptoms of Parkinson’s disease including the hallmark slowness of movement, termed bradykinesia, were described more than 100 years ago. Despite significant advances in elucidating the genetic, molecular and neurobiological changes in Parkinson’s disease, it remains conceptually unclear why exactly patients with Parkinson’s disease move slowly. To address this, we summarize behavioural observations of movement slowness in Parkinson’s disease and discuss these findings in a behavioural framework of optimal control. In this framework, agents optimize the time it takes to gather and harvest reward by adapting their movement vigour depending on the reward that is at stake and the effort that needs to be expended. Thus, slow movements can be favourable when the reward is deemed unappealing or the movement very costly. While reduced reward sensitivity, which makes patients less inclined to work for reward, has been reported in Parkinson’s disease, this appears to be mainly related to motivational deficits (apathy) rather than bradykinesia. Increased effort sensitivity has been proposed to underlie movement slowness in Parkinson’s disease. However, careful behavioural observations of bradykinesia are inconsistent with abnormal computations of effort costs due to accuracy constraints or movement energetic expenditure. These inconsistencies can be resolved when considering that a general disability to switch between stable and dynamic movement states can contribute to an abnormal composite effort cost related to movement in Parkinson’s disease. This can account for paradoxical observations such as the abnormally slow relaxation of isometric contractions or difficulties in halting a movement in Parkinson’s disease, both of which increase movement energy expenditure. A sound understanding of the abnormal behavioural computations mediating motor impairment in Parkinson’s disease will be vital for linking them to their underlying neural dynamics in distributed brain networks and for grounding future experimental studies in well-defined behavioural frameworks.

## Introduction

Parkinson’s disease is a common neurodegenerative disorder defined clinically by the hallmark presence of bradykinesia, i.e. slowness of movements, combined with tremor, rigidity or both^1,2^. The clinical expression of symptoms in individual patients with Parkinson’s disease is very heterogeneous comprising gait problems, autonomic dysfunction, depression, apathy, anxiety and more. Neurobiologically, several neural systems are affected, most prominently midbrain dopaminergic neurons of the substantia nigra pars compacta (SNc)^3^. While there is no curative treatment, several clinically effective symptomatic treatments exist including drugs restoring the neurotransmitter dopamine, electrical stimulation of the basal ganglia termed deep brain stimulation (DBS) and non-pharmacological treatments such as physiotherapy. There has been significant progress in elucidating molecular, genetic and neurobiological changes in Parkinson’s disease ^1^. However, in order to link the findings from increasingly sophisticated experimental techniques on one hand and patients’ clinical impairment on the other, there is a need to better understand the behavioural deficits of patients with Parkinson’s disease ^4^. In particular, we need to grasp the behavioural computations underlying any motor impairment that we wish to improve. In this review, we will summarize observations of the hallmark symptom of Parkinson’s disease, bradykinesia, and consider these observations in a behavioural framework borrowing concepts from optimal control and utility theory^5–7^. In particular, we will discuss how optimal decisions and movements may be based on computations of reward and effort and how this might go awry in Parkinson’s disease.

## Abnormal movement control in Parkinson’s disease

**Figure 1:**
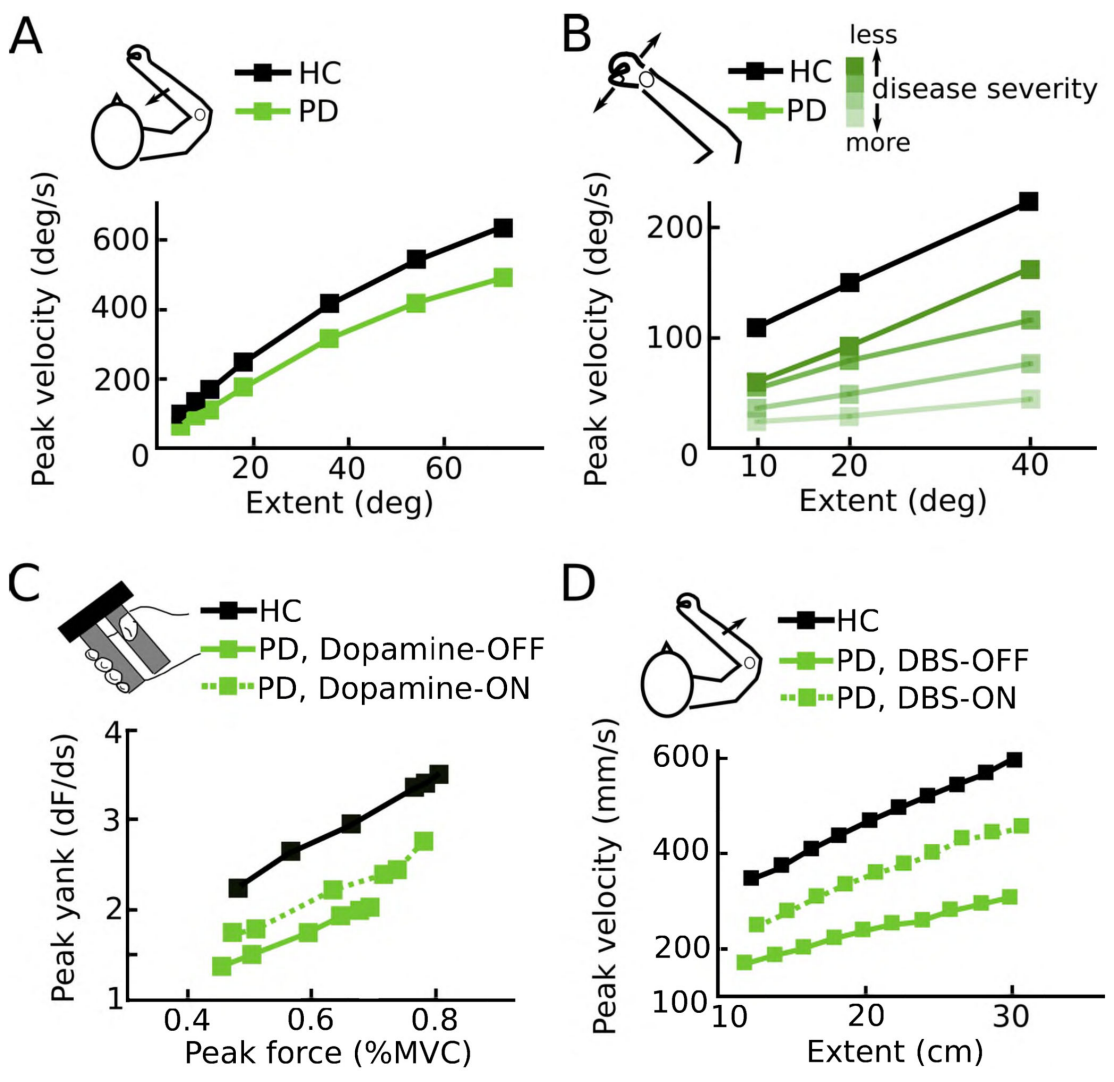
Abnormal vigour in Parkinson’s disease **A.** Parkinson’s disease patients use abnormally low peak velocities for a given movement extent compared to healthy people. Based on male participants from Pfann et al.^11^ studying ballistic flexion movements over the elbow joint **B.** The more severe the disease, as indexed by bradykinesia scores, the lower the velocity that patients on average use for a given extent. Based on the median values from Warabi et al.^12^ studying flexion and extension movements of the wrist **C.** Dopaminergic medication increases the yank that patients exert for a given peak force. Based on Le Bouc et al.^22^ studying a manual gripping task **D.** Likewise DBS can increase the velocity for a given movement extent. Based on Baraduc et al.^8^ studying ballistic extension movements over the elbow joint. DBS, deep brain stimulation; Deg, degrees; dF, change in force; HC, healthy control; %MVC, percentage of maximum voluntary contraction; PD, Parkinson’s disease; s, seconds.

There is a vast literature studying movements in Parkinson’s disease patients. As can be expected from the clinical diagnosis based on the presence of bradykinesia (see box 1 for a glossary), the most consistent finding is that movements are performed abnormally slowly^2^. However, this does not entail that patients fail to express any modulation of movement velocities. Healthy people scale movement velocities by the amplitude of the movement so that e.g. reaching to a target with a distance of 30 cm has a higher peak velocity than a 20 cm reach. Parkinson’s disease patients also scale movement velocities based on amplitudes and are capable of reaching to high distance targets with velocities that are comparable to that of healthy subjects reaching to lower distance targets. In other words, under certain constraints patients are able to reach relatively normal movement velocities. However, they usually express an abnormally flat velocity-to-amplitude slope so that they on average use velocities that are too low for a given amplitude (figure 1A). This impairment is not limited to a certain type of movement but has been demonstrated for a variety of different movements^8–14^. Electrophysiologically, it has been related to an abnormal activation of the agonist muscle in single-joint movements^10,11,15–18^ and impaired coordination of muscle activation in more complex, multi-joint movements^19,20^. During isometric contractions the analogous phenomenon can be observed, namely an abnormally slow change of force (yank) for a given peak force level^21–25^. Interestingly, differences in the amplitude (as opposed to the velocity) of ballistic movement or in the peak force (as opposed to the yank) of isometric contractions between Parkinson’s disease patients and healthy people are less pronounced and less consistently reported unless patients are severely affected^26–29^. Thus, many patients seem able to reach relatively normal amplitudes and force levels, but they only reach this state after a pathologically prolonged movement duration. This impairment becomes progressively worse in more severe disease stages (figure 1B) and can partly be ameliorated by therapies such as intake of dopaminergic medication (figure 1C) or DBS (figure 1D).

Together, this indicates that bradykinesia does not strictly reflect an inability to perform movements with a certain velocity, but that the velocity assigned to a given movement extent (e.g. the amplitude of a reaching movement) is reduced. This has been described as an impaired movement vigour^10,30–33^.

## What is vigour?

**Figure 2:**
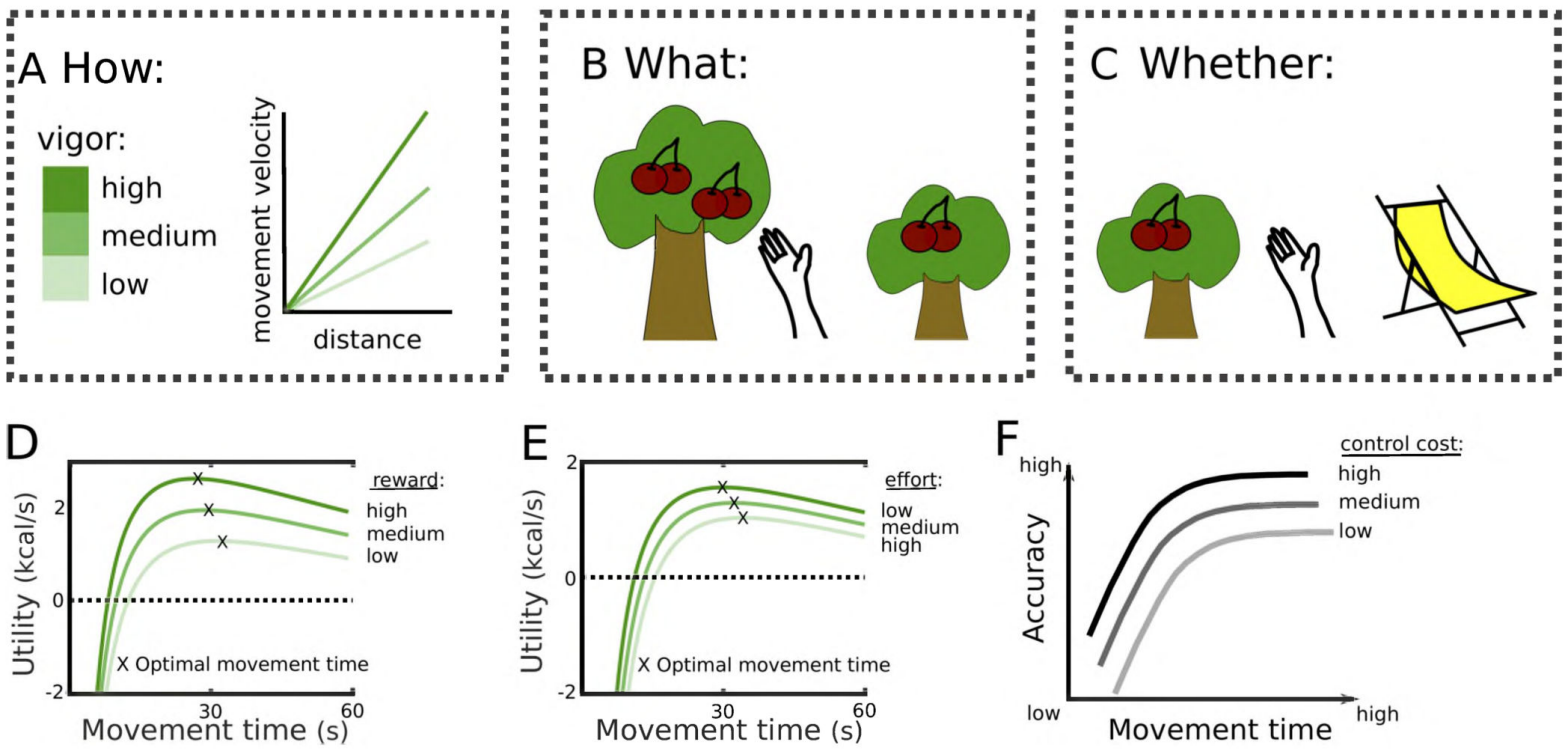
Vigour. **A.** Vigour can be represented by the movement velocity as a function of distance (rather than velocity per se, since velocity scales with distance). The higher a velocity for a given distance (reflected by the steepness of the slope) the higher the vigour. **B.** Another definition of vigour is the propensity to expend effort. This is typically measured by testing whether people prefer options which are effortful (represented by the height of the tree trunk) but lead to higher rewards (4 cherries) or options which are less effortful, but also lead to lower rewards (2 cherries). **C.** This can also be assessed by testing whether people want to engage in a task (sometimes tested by the frequency of reward collection, e.g. how often would they climb a tree to pick cherries) or rather rest (here indicated by a deck chair). **D.** When optimizing utility, increasing the reward can alter the optimal movement vigour. The maximum of the utility as a function of movement time is shifted to the left for high vs. low rewards, indicating that the movement speed that optimizes utility depends on the reward that can be acquired. **E.** The same can be observed when altering the effort costs of the movement. The equations and parameters for the plots in D&E are based on Shadmehr and Ahmed^5^ and correspond to caloric expenditure during a 50 m walk. **F**. Fast movements are related to lower accuracy. This relationship can be modulated through increases in control to reduce noise and thus improve accuracy ^47^. Throughout the figure the level of vigour is indicated by the opaqueness of the green colour.

The term vigour is often used somewhat vaguely and there is considerable heterogeneity in the way it is defined. The definition that most closely relates to the deficit observed in Parkinson’s disease is the velocity of a movement for a given movement extent^34^, see figure 2A. This definition is close to our intuitive understanding describing *how* a movement is expressed, determining the time it takes to reach the goal of an action (box 1). Vigour has also been defined as the propensity to exert effort when deciding *what* to choose or *whether* to engage in a task at all (figure 2B & C) putting modulation of vigour primarily in the context of decision-making rather than motor control. Even though decision-making and motor control are mainly studied in separate fields they share many commonalities including the computations underlying vigour modulations^5,7,35,36^.

An important modulator of vigour is the subjective value or utility that can be obtained when pursuing goals (box 1). The utility of an option does not only affect whether it is chosen but also how it is retrieved. Shadmehr, Ahmed and colleagues have developed a normative framework for vigour modulations during value-based decisions^5–7^. Like optimal foraging theory, this framework postulates that animals make decisions by computing a utility function that depends on the reward that can be obtained subtracted by the effort that has to be spent and divided by the total time it takes to acquire and harvest the reward. When considering caloric intake, the utility reflects the energy obtained (acquired minus spent) per unit time and is thus equivalent to the capture rate^37^. However, the term reward here refers to any incentivizing value, such as food, water or money (see also box 1). Ahmed and Shadmehr extended this framework by postulating that decision-making is not solely concerned with computations of subjective values for different rewards but also *how* these should be acquired. Since the movement vigour will both affect the effort that has to be spent and the time it takes to gather and harvest the reward, it directly feeds into the computation of utility. In particular, slow movements can be ‘costly’ despite being closer to the optimum regarding energy consumption, since reward acquisition is delayed (reward is discounted in time) and the increased time to gather the reward constitutes an opportunity cost (no other rewards can be obtained). This model predicts that increasing the utility e.g. by increasing the reward value (figure 2D) or reducing the effort cost (figure 2E), should result in increased movement vigour, which has been demonstrated in several studies^38–41^.

Effort costs are not only related to movement energy expenditure as described above, but also neural costs and the requirement of resources for a specific movement^42–44^. Furthermore, what can also be considered a cost of fast movements is the deterioration of movement accuracy, which is termed speed-accuracy trade-off^45^. This is because faster movements require a larger motor command, which increases motor noise^46^, and accuracy can only be preserved at the cost of increased control in order to reduce this noise^47,48^, see figure 2F.

In summary, modulations of vigour can be conceptualized as a process optimizing which options to choose and the time we should take to reach a certain goal by considering the reward that can be acquired given the associated costs such as missed opportunities and effort costs. Could abnormal computations of rewards or costs underlie bradykinesia in Parkinson’s disease?

## Effects of reward and effort on vigour in Parkinson’s disease

**Figure 3:**
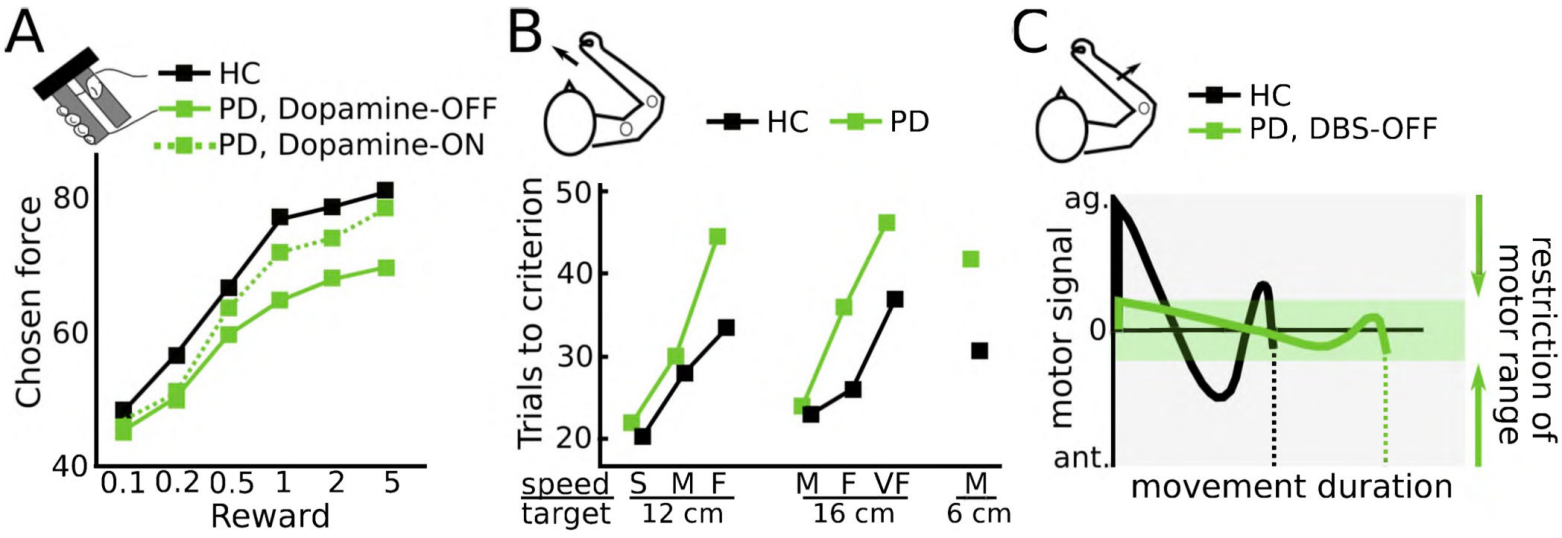
Effects of reward and effort on movement in Parkinson’s disease. **A.** Parkinson’s disease patients express an abnormally flat slope between exerted force and reward resulting in lower force levels when rewards are high. This reduced ‘reward-sensitivity’ is improved by dopamine and mainly observed in patients with apathy. Based on Le Bouc et al.^22^ studying a manual gripping task during varying levels of reward. **B**. Parkinson’s disease patients are able to reach targets with a certain required velocity (S, slow; M, medium; F, fast; VF, very fast) like healthy people, but need a larger number of trials. This is particularly pronounced when the movement demands acceleration and deceleration in close succession (compare e.g. reaching with fast (F) velocities to a 12 cm vs. 16 cm distant target). Based on Mazzoni et al.^31^ studying reaching movements. Copyright 2007 Society for Neuroscience. **C.** Using a computational model Baraduc and colleagues showed that Parkinson’s disease patients show an abnormally narrow motor range resulting in long movement duration despite otherwise normal movement parameters. Based on Baraduc et al.^8^ studying ballistic extension movements over the elbow joint. ag, agonist; ant., antagonist; DBS, deep brain stimulation. HC, healthy control; PD, Parkinson’s disease.

Several studies have assessed how Parkinson’s disease patients choose among options with varying levels of monetary reward and effort costs. Le Heron and colleagues^49^ tested Parkinson’s disease patients with and without apathy, ON and OFF dopaminergic medication and a healthy control group performing a reward-effort trade-off task. Participants could accept or reject offers where different levels of isometric grip-force had to be produced in order to obtain different amounts of monetary reward. While apathy mainly reduced acceptance rates for low-reward offers, dopaminergic medication had a different effect, increasing the acceptance for high reward, high effort options. In a related study Le Bouc et al.^22^ investigated the reward-effort trade-off in Parkinson’s disease patients ON and OFF dopaminergic medication and a healthy control group using two tasks. First, in an incentive force task, patients produced grip forces, which were multiplied by varying levels of monetary reward shown on the screen. Second, in a binary choice task, participants chose between a low reward - low effort option and a variable high reward - high effort option. Effort was reflected by the required peak grip force. The high reward - high effort option was adjusted in a staircase procedure resulting in equivalence levels between effort and reward. As expected, irrespective of monetary incentives patients OFF medication used a significantly lower yank for a given force level compared to patients ON medication and healthy people (shown in figure 1C). Furthermore, the effort (in this study defined as peak force) participants were willing to exert for higher rewards was significantly lower in patients OFF medication compared to ON medication and healthy people in both tasks, see figure 3A. Thus, patients were effectively less willing to produce higher forces for higher rewards. To further disentangle these effects, Le Bouc et al. used a computational model that incorporated several free parameters including reward sensitivity and motor activation rate (the latter parameter reflected how neural drive results in muscle activation). Using model comparison, they found that in the model that best explained the data, dopamine modulated both the motor activation rate and reward sensitivity. However, these parameters had distinct effects on the observed behaviour. A reduction in motor activation rate in patients OFF medication reflected the observed flattened yank-force slope (corresponding to our definition of movement vigour, see figure 1C & 2A) irrespective of reward and correlated with patients’ bradykinesia scores. In contrast, reductions in reward sensitivity reflected the slope between reward and effort (figure 3A) and correlated with patients’ apathy scores rather than bradykinesia severity. Thus, while reduced reward sensitivity has repeatedly been reported in Parkinson’s disease patients ^22,49–55^ it appears to be more closely related to impaired motivation (apathy)^56,57^, arguing against a central role of abnormal reward sensitivity underlying bradykinesia. Further evidence for distinct mechanisms underlying motivational and movement deficits comes from the clinical observation that Parkinson’s disease patients can loose their motivation e.g. to pursue their hobbies and engage in activities despite significant improvement in bradykinesia after DBS surgery^58,59^.

### Could increased sensitivity to effort costs underlie bradykinesia?

Mazzoni and colleagues studied seven patients with Parkinson’s disease ON medication^31^. Patients had to perform reaching movements to targets with varying distances. While feedback regarding their endpoint-accuracy was given, the critical determinant of trial validity depended on whether the velocity fell within a specified range. Patients had to complete twenty trials of each trial type (different combinations of distances and velocities) in order to successfully finish the experiment. The main result from the study was that patients were able to successfully complete the task but required a larger number of trials (termed ‘trials to criterion’) compared to healthy participants, see figure 3B. In other words, the range of possible movement velocities was similar to that of healthy controls, but patients were more likely to use lower velocities. Parkinson’s disease patients needed particularly many trials when these required a high amount of velocity changes for a given movement duration (e.g., fast movements to close targets require large acceleration and deceleration in close succession), which was measured as the average absolute acceleration. This measure correlated with the energetic cost of the movement (power expenditure of the arm) and with the patients’ clinical motor impairment. Since in this study patients were able to expend the required energetic movement cost, but were less likely to do so (i.e. need a higher number of trials), it was concluded that slow movements in Parkinson’s disease were due to an increased sensitivity to energetic movement costs rather than an inability to exert this effort. Another important finding was that patients and healthy controls did not differ regarding their movement accuracy or other kinematic parameters arguing against a speed-accuracy trade-off underlying bradykinesia. This finding is in line with several other studies reporting similar kinematic measures such as movement trajectories, variability and end-point accuracy in Parkinson’s disease patients and healthy people^16,24,29,60,61^. For example, Baraduc et al.^8^ tested Parkinson’s disease patients performing reaching movements to varying distances without visual feedback. As illustrated in figure 1D, patients showed abnormally low vigour, i.e. low velocities for a given distance, which could be ameliorated through therapeutic DBS. The authors then analysed the observed data using a model of optimal motor control in which motor commands are transformed to kinematics using a set of differential equations (see Baraduc et al.^8^ for more details). The optimal motor command yielded the movement trajectory that minimized the neuromuscular cost of the movement given movement amplitude and duration. This analysis showed that the same model was able to account for the movements of patients and healthy people, but that the groups differed regarding their range of motor commands (which in this study corresponded to the range from baseline to maximal population motoneuron activity, similar to the motor activation rate during isometric contractions in Le Bouc et al.^22^). This range was abnormally narrow in Parkinson’s disease patients leading to slower movement velocities despite otherwise normal model parameters, see figure 3C. In other words, given their abnormal motor command range, patients performed optimal (i.e. cost minimizing) movement trajectories suggesting that except from abnormally low vigour, patients are able to execute normal movements. Thus, the results of these studies argue against increased control costs to account for impaired movement accuracy underlying bradykinesia and are in line with increased sensitivity to movement energetic costs^8,31,62^. Another way to put this is that Parkinson’s disease patients might have reduced implicit motivation ^31,63^ (since it is controlled outside of awareness, in contrast to explicit motivation) to expend energy for selected movements. This would result in abnormally slow movements unless patients are explicitly motivated by extrinsic factors such as task instructions^31^ or urgency^64^. For example, a patient who is immobilized by their disease might suddenly be able to move rapidly when faced with immediate danger, e.g. running out of a burning house, a phenomenon termed paradoxical kinesia ^64,65^. Thus, the lack of implicit motivation causing bradykinesia due to reduced energy invested in movement may be overcome by significant extrinsic motivation. Interestingly, this energy cost account of bradykinesia resembles a hypothesis that has been put forward over 40 years ago, ascribing bradykinesia to an impaired ‘energization’ of movement^10^. Yet, there are observations that argue against bradykinesia being related to energy preservation.

## Is bradykinesia caused by reduced willingness to energize movement?

**Figure 4:**
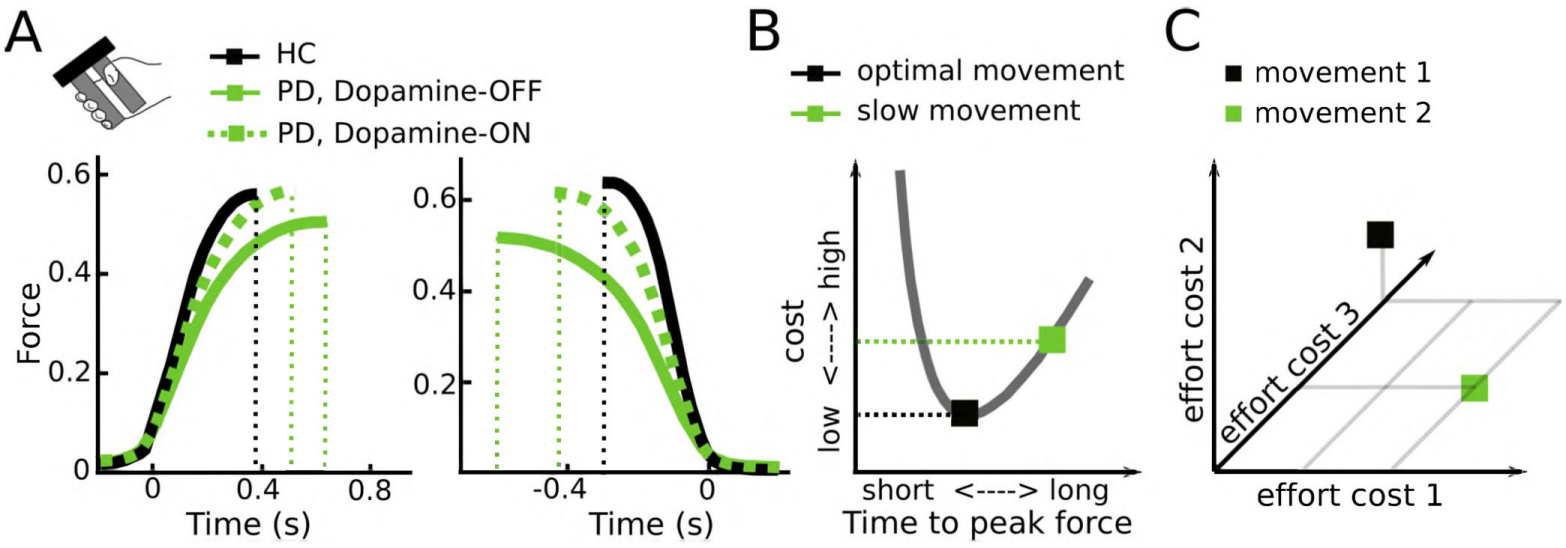
Effort costs of movement. **A.** Parkinson’s disease patients do not only show an abnormally slow increase in force (positive yank, left column), but also an abnormally slow decrease in force (negative yank, right column) leading to larger absolute force that is exerted (area under the curve) even if the peak force is lower. Based on Le Bouc et al.^22^ studying a manual gripping task. **B**. Since people usually use movement durations near the optimum regarding its energetic cost, slowing down movements results in increased costs. The cost here reflects the instantaneous movement cost (neural drive leading to muscle activation) integrated over the movement duration. Based on Le Bouc et al.^22^ **C.** Movements are presumably related to a composite cost consisting of different effort costs, here shown for three dimensions. For example, movement 1 (black) has higher costs regarding effort 2 and 3, but lower costs regarding effort 1 compared to movement 2 (green) and Parkinson’s disease patients might be impaired in exerting a particular effort cost altering the composite cost compared to healthy people. HC, healthy control; PD, Parkinson’s disease.

People tend to move at velocities near the optimum regarding energy consumption^5^. Not only moving faster, but also moving slower can increase energy expenditure, since the metabolic cost increases linearly with movement duration^5,66^. The very slow movements observed in Parkinson’s disease will therefore not reduce, but increase the energetic cost of the movement. In line with this, Parkinson’s disease patients spend more energy when walking compared to healthy people^67^. Furthermore, rigidity, an increased resistance to passive movement perceived as muscle stiffness and another hallmark motor symptom of Parkinson’s disease ^1,68,69^, comes at the cost of increased energy expenditure. Finally, during isometric contractions Parkinson’s disease patients do not only show a reduced activation rate (positive yank) but also strongly reduced relaxation rate (negative yank), which has been demonstrated in a multitude of studies^27–29,70–73^. For example, in the study by Le Bouc et al.^22^ discussed above both activation and relaxation rate in Parkinson’s disease patients were abnormally low particularly OFF dopaminergic medication (figure 4A), increasing the energetic cost of the movement (figure 4B) even if the exerted peak force is slightly lower. An increase in the absolute exerted force has also been observed in Parkinson’s disease patients performing externally-paced isometric contractions^74^ and precision grips^75,76^. Together, these observations indicate that bradykinesia does not necessarily reduce the energy invested in movements.

Another challenge of the energy-preservation account of bradykinesia is the assumption that patients could use higher velocities if they were ‘willing’ to do so. For example, Mazzoni et al.^31^, observed that Parkinson’s disease patients were able to reach targets with the required velocity but needed a larger number of trials. An explanation for this could be, that the chosen vigour (velocity for a given movement extent) assigned to a movement is probabilistic and that the probability distribution is shifted to lower velocities in Parkinson’s disease. Irrespective of the exact mechanisms underlying this shift, drawing more samples from this distribution will lead to sufficient trials with the required velocity at some point. While the authors addressed this to some extent by computing (non-normalized) probability distributions, and the seven included patients all were able to reach the required velocities it is questionable whether a patient with severe bradykinesia OFF medication would be able to do so. Finally, it is important to note that, while motor function in Parkinson’s disease can fluctuate strongly, the occurrence of paradoxical kinesia is rare and most patients will not have experienced this phenomenon even when faced with immediate danger e.g. in war times^77^. The more common mild urgency-related improvement in bradykinesia might be more closely related to general arousal^78^ and, similar to the well-known beneficial effects of external cueing^64,79–81^, might be less dependent on SNc innervation of the basal ganglia^78^.

So why are Parkinson’s disease patients slow if not due to reduced willingness to expend energy? Bradykinesia could still be in line with an inability to exert effort (or increased effort sensitivity), but this effort might not be simply reflected by a (directly measurable or computable) change in movement energy. The observation that patients are also impaired in relaxing a contraction^27–29,70–73^, halting, correcting, or decelerating a movement^71,82,83^ suggests that motor impairment in Parkinson’s disease includes a general impairment in transitioning between stable and dynamic movement states. While stabilizing the current body position can be useful, e.g., when stabilizing body posture against external perturbations to prevent falls, it can become detrimental when it needs to be changed during or in anticipation of voluntary movement. Be it a crouching tiger attacking its prey, a sprinter commencing from the starting block or simply a person releasing a firm handshake this transition is central to physiological motor control. A bias towards the stable state is also consistent with the observed rigidity in Parkinson’s disease, i.e. muscle co-contractions that reinforce a postural state^69^. The processes underlying the shift between stable and dynamic motor states could be viewed as another effort cost to be evaluated when determining the vigour of an intended action; in addition to efforts reflecting specific properties of a movement (such as required movement energy or end-point accuracy). It is currently not well-known how the brain computes effort costs based on e.g. neural energy demands and allocation of resources in contrast to more easily computable movement energy costs^48,84^. However, it seems likely that the brain computes a composite cost reflecting different efforts for decision-making and motor control^42–44,46^, illustrated in figure 4C. Parkinson’s disease patients might be particularly impaired in exerting an effort that does not directly reflect movement energy altering the composite effort related to movement compared to healthy people.

It seems difficult to disentangle whether Parkinson’s disease patients have increased sensitivity to computed effort or are impaired in exerting it, because their effects on behaviour would be indistinguishable in most cases. However, there are clinical observations arguing for the latter. Parkinson’s disease patients do not only move more slowly compared to healthy people, but this deteriorates over time during repetitions (e.g. finger tapping) so that movements become slower and smaller (termed decrement or sequence effect^2^). This characteristic phenomenon of Parkinson’s disease^85^ is unlikely to be related to abnormal effort computations, since this would require the computation of separate (increasing) effort costs for each iteration of repetitive movements. Ultimately, both increased effort sensitivity and difficulties in its implementation might contribute to bradykinesia. Elucidating which exact efforts are particularly costly or difficult to exert for Parkinson’s disease patients will be a crucial step in better understanding motor impairment in Parkinson’s disease.

## Outlook

**Figure 5:**
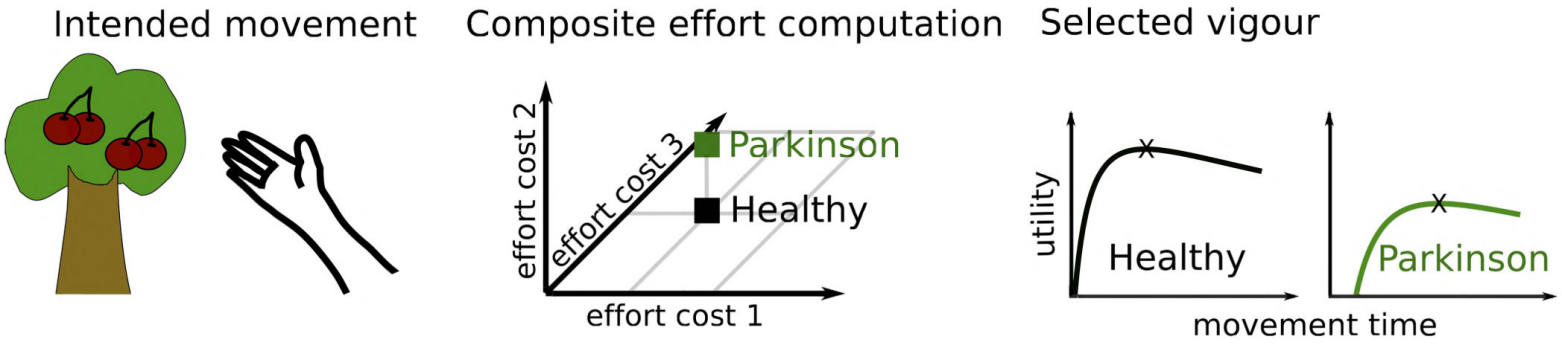
Summary To optimize movement vigour an agent has to take into account the reward that can be obtained and the different effort costs (such as muscular energy expenditure, motor state transitions, resource allocation, accuracy requirements, etc.) that need to be expended to reach the goal. An increased composite effort cost in Parkinson’s disease can lead to abnormally slow movements despite otherwise normal (i.e., utility optimizing) motor control mechanisms.

In this paper, we discussed how bradykinesia could result from abnormal utility computations based on the rewards and efforts associated with an action (summarized in figure 5). Behavioural observations mainly suggest that Parkinson’s disease patients move slowly because of an abnormal computation of, or an inability to exert, a composite effort reflecting the disparate costs incurred by the transitioning to and from a dynamic movement state and by the execution of specific movements themselves. The motivation for this review was to discuss behavioural accounts of bradykinesia in terms of the putative computations underlying the observed movement deficits borrowing concepts from optimal control and utility theory. It should be noted that Parkinson’s disease patients suffer from a variety of other debilitating symptoms including anxiety, depression, cognitive dysfunction and more, which are beyond the scope of the current review.

A sound understanding of the behavioural impairment in Parkinson’s disease is vital for therapeutic advances. While the effect of dopamine on reward computations has been studied quite extensively, we hope that this review sparks interest in further delineating how alterations in distinct effort costs might affect motor impairment in Parkinson’s disease. Beyond the general implications for improving our understanding of patients’ symptoms, this might also contribute to improving therapy. In particular novel therapeutic techniques, such as adaptive DBS, which adapts stimulation to changes in patients’ clinical states ^86–89^, offer the opportunity to directly target specific neural signals. To this end it will be vital to link the behavioural impairment to changes at the neural implementation level. There has been significant progress in our understanding of the (physiological and pathological) implementation of reward- and effort-based movement control in cortical and subcortical networks of the brain^5,90^. For example, there is a wealth of studies linking midbrain dopaminergic innervation of cortico – basal ganglia networks (in particular ventral tegmental area and ventral striatum) to reward prediction errors, action outcomes and value^91–94^. Even though several studies have demonstrated a gradient from ‘reward’-related computations in more ventral loops to movement-related activity in more dorsal (SNc and dorsal striatum) loops^95–97^ kinematic correlates have also been recorded in ventral areas^98–100^ and it remains to be elucidated how exactly this is altered in Parkinson’s disease^101,102^. Given the functional architecture of the basal ganglia and the effects of dopamine release on neural excitability and plasticity^30,103–105^, cortico – basal ganglia networks would be well-suited to mediate vigour modulations^30,33,35,106,107^. This might both be possible through gain modulation of downstream pre-motor areas^30,106^ or feedback connections to cortical areas thought to be involved in effort cost computations and evaluations^57^. Furthermore, abnormal modulation of certain oscillatory frequency bands (in particular the 13-30 Hz beta band in the subthalamic nucleus) has been demonstrated in Parkinson’s disease. Since this is strongly modulated prior to and during movement, related to patients’ motor impairment and reduced by therapeutic dopaminergic medication and DBS, it is a strong candidate neural feedback marker for adaptive DBS. There is evidence that beta activity more closely reflects the transitioning between stable and dynamic movement states than movement energetic costs^108–110^. Thus, restoring physiological beta activity modulation in Parkinson’s disease might improve patients’ ability to flexibly adapt their behaviour. Finally, dynamical systems theory has made significant contributions to linking multidimensional cortical population dynamics to movement control over the last years ^111^. This framework might be particularly suited for studying motor impairment in Parkinson’s disease, since it is concerned with the relationship between neural state transitions and behaviour ^111,112^. Basal ganglia architecture resembles a recurrent negative feedback system^106^. Such systems are particularly suited for stabilizing (cortical) attractor states and allowing state changes when necessary, e.g. during purposeful movement^48,113^. Recently cortical neural ‘null’ spaces have been proposed to allow movement preparation without overt movement^114^. In order to allow muscle activation during movement execution the neural trajectories transition to ‘output-potent’ spaces^114^. Thus, these neural state transitions might resemble the stable vs. dynamic movement states discussed above. It remains to be shown whether dynamical systems theory could be a helpful framework for improving our understanding of the neural basis of motor impairment in Parkinson’s disease.

There is an ever-increasing wealth of studies elucidating neural activity and network patterns in cortico-basal ganglia networks and their modulation by midbrain neurotransmitter systems. To avoid widening the gap between our understanding of neural dynamics and their effects on behaviour, careful examinations of behaviour and the underlying computations will be vital for advancing Parkinson’s disease research. Grounding experimental studies in well-defined behavioural frameworks will allow us to gain important insights into physiological control of movement and how this might go awry in patients with Parkinson’s disease and other brain disorders.

## Data Availability

This review article does not include any newly acquired data.

## Box 1 Glossary

### Bradykinesia

The direct translation of bradykinesia is slow movement. Strictly, the related terms akinesia and hypokinesia reflect, respectively, fewer or no movements and movements of reduced amplitude, but the terms are often used interchangeably^115^. In the clinical assessment bradykinesia scores also incorporate movement interruptions, hesitations and amplitude decrement^116^. Here, we use bradykinesia in its general sense as slowness of movement.

### Dynamical systems theory

A mathematical description of complex dynamical systems. For example, the behaviour of a pendulum can be described by a two-dimensional dynamical system with the state variables position and velocity. This approach can be extended to high-dimensional data and has been successfully applied for analysing simultaneous recordings of large populations of neurons during movement^111^.

### Effort

The subjective work that must be exerted in order to achieve a goal. The overall effort assigned to an action is presumably not given by a single quantity, but consist of different costs, such as muscular energy, demand for resources and accuracy requirements^42–44,46^.

### Reward

The term reward has often been used in a rather general and vague way in Neuroscience comprising reinforcer, appetite or pleasure^117^. In models of basal ganglia function reward is mostly used in terms of computations of incentivizing values e.g. of nutrient or money, that are closely related to the concept of utility (see below) and often contrasted to ‘motor’ functions solely concerned with movement. This can be problematic because, apart from general issues with putative value computations in the brain^118^, the contrast between reward and action is somewhat artificial in that computations of reward are only helpful if they inform our behaviour, i.e. our actions^119^. Here, we use the term reward for (explicit) incentivizing values, which in the reviewed Parkinson’s disease studies corresponds to money or collected points.

### Utility

A representation of the usefulness or desirability of an object, which amongst others depends on its value, probability of gain and the state of the actor^120,121^. In optimal foraging theory utility is equal to the capture rate which is given by the obtained energy subtracted by the spent energy and divided by time^37^. The actor can then decide between exploring and exploiting a current patch by comparing the local (current) capture rate with the global capture rate. In general, utility theory ranks different objects based on an individual’s choices.

### Vigour

In everyday speech vigour is often used to described bodily or mental force, or the intensity of action. In Neuroscience the term has been used variably including the force, speed, amplitude and frequency of actions. The definition that we here use for movement execution is the velocity for a given movement extent (because velocity scales with movement extent such as amplitude, see figure 2A), which at its core optimizes the time that we need to reach the goal of a movement. This is central to optimizing the utility and effort cost in value-based decision-making and motor control theory^5–7^ and crucial for allowing skillful movement^30^. Vigour can also be defined in the context of decision-making as the ‘willingness’ to work harder or longer (e.g. should I collect a reward which requires a lot of effort, or choose the alternative which is less rewarding but easier to collect), or the propensity to work at all (as compared to resting), see figure 2 B&C.

## Abbreviations

DBS: deep brain stimulation
SNc: substantia nigra pars compacta
